# Effects of Sterilization and Hydrolytic Degradation on the Structure, Morphology and Compressive Strength of Polylactide-Hydroxyapatite Composites

**DOI:** 10.3390/ijms231810454

**Published:** 2022-09-09

**Authors:** Mirosław Kasprzak, Agnieszka Szabłowska, Agata Kurzyk, Paulina Tymowicz-Grzyb, Adrian Najmrodzki, Anna Woźniak, Agnieszka Antosik, Joanna Pagacz, Piotr Szterner, Andrzej Plichta, Piotr Wieciński, Paulina Rusek-Wala, Agnieszka Krupa, Przemysław Płociński, Karolina Rudnicka, Monika Biernat

**Affiliations:** 1 Biomaterials Research Group, Łukasiewicz Research Network–Institute of Ceramics and Building Materials, Center of Ceramic and Concrete in Warsaw, Cementowa 8, 31-983 Kraków, Poland; 2 Faculty of Chemistry, Warsaw University of Technology, Noakowskiego 3, 02-677 Warsaw, Poland; 3 Department of Immunology and Infectious Biology, Faculty of Biology and Environmental Protection, University of Łódź, Banacha 12/16, 90-237 Łódź, Poland; 4 Bio-Med-Chem Doctoral School, University of Lodz and Lodz Institutes of the Polish Academy of Sciences, Banacha 12/16, 90-237 Łódź, Poland

**Keywords:** biocomposites, hydrolytic degradation, sterilization, PLA, hydroxyapatite

## Abstract

Composites based on polylactide (PLA) and hydroxyapatite (HA) were prepared using a thermally induced phase separation method. In the experimental design, the PLA with low weight-average molar mass (*M*_w_) and high *M*_w_ were tested with the inclusion of HA synthesized as whiskers or hexagonal rods. In addition, the structure of HA whiskers was doped with Zn, whereas hexagonal rods were mixed with Sr salt. The composites were sterilized and then incubated in phosphate-buffered saline for 12 weeks at 37 °C, followed by characterization of pore size distribution, molecular properties, density and mechanical strength. Results showed a substantial reduction of PLA *M*_w_ for both polymers due to the preparation of composites, their sterilization and incubation. The distribution of pore size effectively increased after the degradation process, whereas the sterilization, furthermore, had an impact on pore size distribution depending on HA added. The inclusion of HA reduced to some extent the degradation of PLA quantitatively in the weight loss in vitro compared to the control without HA. All produced materials showed no cytotoxicity when validated against L929 mouse skin fibroblasts and hFOB 1.19 human osteoblasts. The lack of cytotoxicity was accompanied by the immunocompatibility with human monocytic cells that were able to detect pyrogenic contaminants.

## 1. Introduction

Bone damage, whether generated by congenital disorders, diseases, trauma or accidents, depicts a significant burden on society and the health system [1,2]. One of the most frequent metabolic disorders is osteoporosis which might affect over 23 million people in Europe nowadays [3]. According to International Osteoporosis Foundation, osteoporotic fractures will be experienced by 1 in 3 women and 1 in 5 men over the age of 50. Osteoporosis is characterized by a reduced density of mineral bone caused by a deterioration of bone tissue micro-architecture and loss of bone weight [4]. Other factors influencing bone quality is the age and accompanying diseases such as diabetes [5]. The self-healing of bone is determined by a number of factors such as the quality of the patient’s bone, the size of the gap to the bridge or the stability of the fracture site [6]. The challenges related to bone loss and its regeneration are expected to rise in the future [6]. Therefore, the development of biomaterials for medical implants is continuously evolving in order to enhance their performance to address patients’ needs. The focus is laid not only on the similarities to human bone but on the design the material that can replace the currently used metallic implants in order to avoid another surgery to remove the metal inclusion.

By reason of the fact that bone has the ability to regenerate, the developed biomaterials used as substitution or replacement should possess appropriate resorbable and bioactive properties; these would improve the regeneration of host tissue and consequently replace the implanted biomaterial with newly created bone tissue [7]. As hydroxyapatite (HA) is a natural component of bone, its synthesis method brought a lot of attention due to its product similarity and excellent biocompatibility with natural tissue.

Biological apatite is a nanocrystalline multi-substituted carbonated HA with unique physicochemical and biological properties [8]; this is due to its micro-architecture and composition that vary depending on the type of bone and physiological state of the individual body. Mineralized tissues contain a number of trace elements that substitute constituents in bone substance tissue and its crystals, such as F and Sr present in the solid mineral phase or Si, Cu allocated in the organic matrix or Zn placed in both the solid mineral phase and/or organic matrix [9,10]. Having a similar structure, the synthetic HA is, furthermore, non-toxic and displays biocompatible and osteoinductive properties. Thus, a number of studies started to apply doping of metals into the structure of HA during the synthesis of apatite in order to enrich the functionality of the final product. Study by Ofudje et al. [11] showed that the synthesized nanohydroxyapatite (nHA) with doped Zn had antibacterial properties. Similarly, Gayathri et al. [12] investigated the nHA with substituted magnesium showed antibacterial and larvicidal activity. In addition, recent research by Harrison et al. [13] reported the manufactured nHA with strontium substitution further incorporated into a gel that showed superior biocompatibility and improved radiopacity.

As natural HA occurs in nanoform, the majority of recent studies have been dedicated the HA synthesis and modification of its nanostructure; it is, furthermore, because there is a relationship between the physical properties of HA particles and the ensuing immune response. A study by Lebre et al. [14] showed that smaller needle-shape HA particles generated an extended inflammatory response than the spherical and larger-sized HA particles. A detailed study of needle microsized HA with inclusion of varied metals might bring a new light on the relation between a structure, mechanical properties or biological response. The microsized whisker HA shows in general a greater impact on mechanical properties of HA-rich composites compared to nHA-rich composites [15].

Besides the fact that HA is the main inorganic fraction in the bone extracellular matrix, the primary constituent of the organic matrix is collagen; its primary function is mechanical support acting as a scaffold for bone cells [16]. Biodegradable synthetic polymers such as polyglycolide, poly-(lactide-co-glycolide)s, polyhydroxyalkanoates, polycaprolactone, polylactide (PLA) have been studied as candidates for implant scaffolds showing promising biomedical applications [17]. However, these pure biomedical polymers might not always mimic the mechanical properties of native tissues such as elasticity or modulus because of their intrinsic shortcomings. However they can be used to design structural integrity and mechanical support environment which is favourable for cell attachment, proliferation or differentiation [18]. Among the synthetic polymers, PLA is often repeatedly singled out as the matrix material for small screws and pins among the commercialized bioresorbable implants [19].

The process of designing the polymer-based composites with HA inclusion should consider how material will be sterilized and whether the sterilization will cause any significant changes in the physical, chemical, mechanical and biocompatibility properties of the final product that might generate deplorable responses in the human body or compromise its function [20]. An equally important aspect of composite characteristics, is its degradation pattern on the porosity and mechanical strength in timespan. Besides the large number of research undertaken for synthetic polymers such as PLA in regenerative medicine, only a few studies investigate how sterilization and degradation effect physicochemical properties of manufactured materials with different weight-average molar mass (*M*_w_) of PLA or HA morphology included [21,22,23,24,25,26,27,28].

The aim of this work was to investigate the effect of sterilization and in vitro hydrolytic degradation on the structural and mechanical properties of porous composites prepared from two different *M*_w_ PLA with the inclusion of HA hexagonal rods or whiskers with and without metal addition. To the best of our knowledge, there is no literature reports combining the effect of polylactide molecular properties with varied morphological inclusion of hydroxyapatites on the monitored change in mechanical, physical and chemical properties of composites when sterilized and degraded in time.

## 2. Results

### 2.1. Microstructure of HA

**Figure 1:**
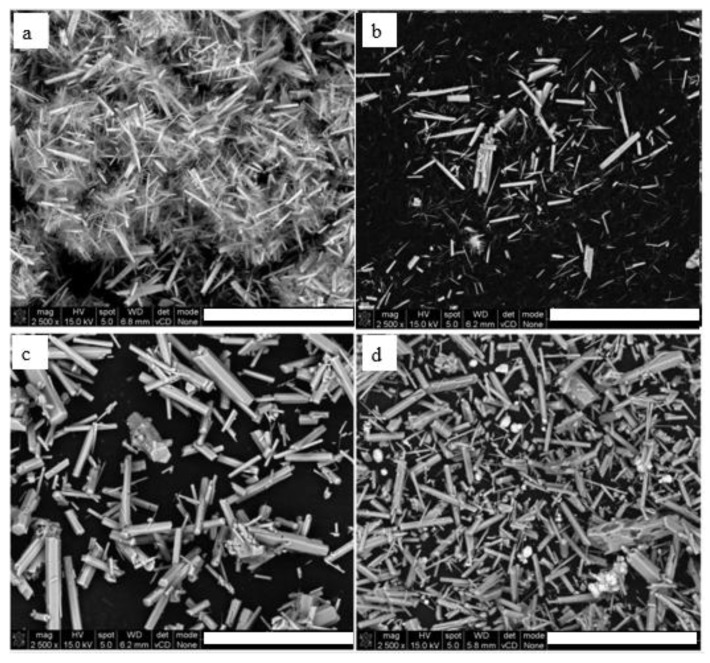
Microstructure of HA: (**a**) whiskers (**b**) whiskers doped with Zn, (**c**) hexagonal rods, (**d**) hexagonal rods mixed with Sr. Scale bar 50 µm.

**Figure A1:**
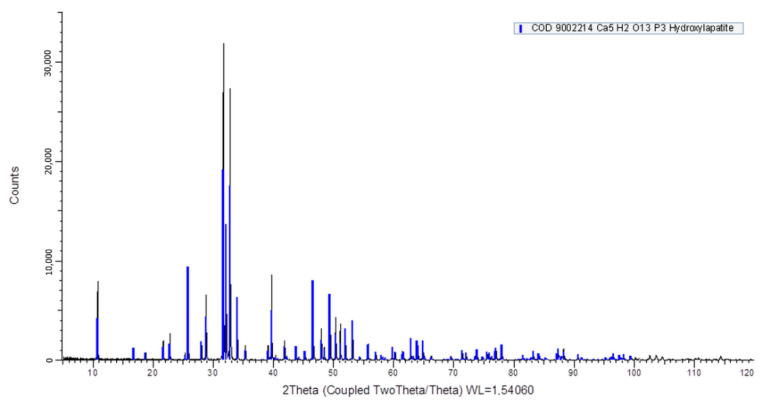
XRD analysis of whiskers.

**Figure A2:**
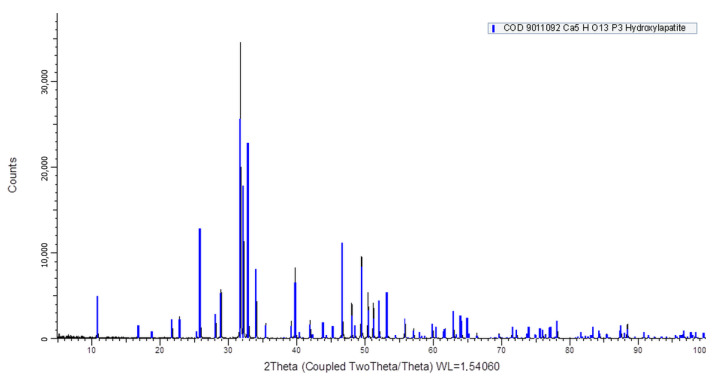
XRD analysis of hexagonal rods.

**Figure A3:**
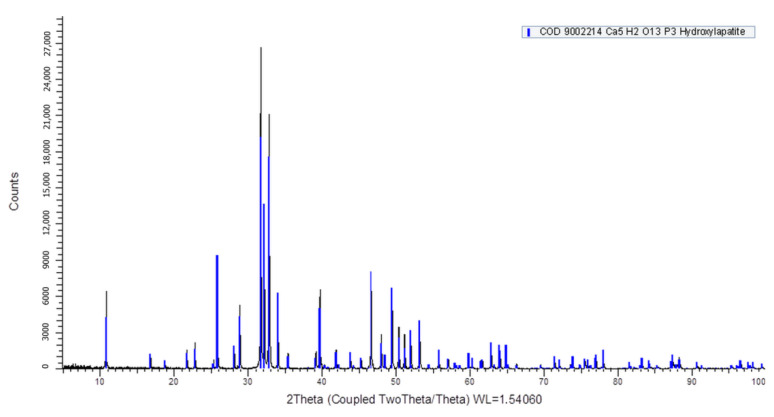
XRD analysis of whiskers with Zn.

**Figure A4:**
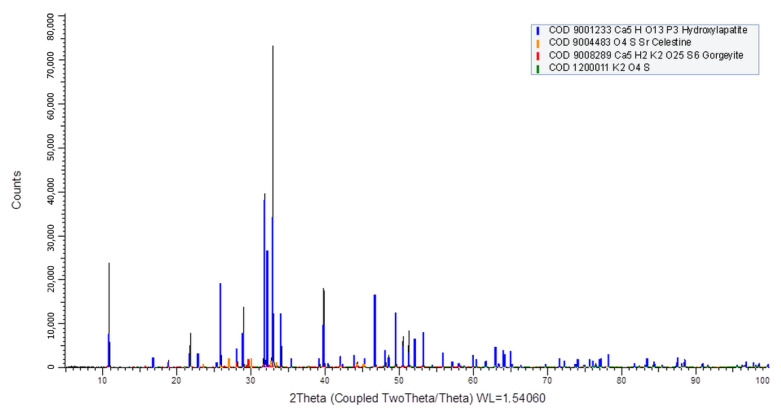
XRD analysis of hexagonal rods with Sr.

The syntheses of HA by homogenous precipitation and chelate decomposition resulted in microsize whiskers and hexagonal rods, respectively (Figure 1a,c). Whiskers had a length of 10–20 µm and a width of 0.2–2 µm, whereas rods showed a length of 5–40 µm and a width of 0.5–3 µm. The XRD analysis confirmed that those materials were mainly composed of HA. Only in the case of hexagonal rods mixed with Sr besides HA, the diffraction peaks corresponding to SrSO_4_, K_2_SO_4_ and K_2_SO_4_×5CaSO_4_×H_2_O phases were identified (Figure A1, Figure A2, Figure A3 and Figure A4).

Modification of HA whiskers by doping Zn ions led to a change of microstructure proved by reduction of length down to 2–10 µm and width 0.01–2 µm (Figure 1b). The efficiency of modification was confirmed by EDS analysis. The HA in the form of hexagonal rods with Sr was prepared by mixing Sr salt with HA. After this modification, the Sr ions were not built into the HA structure, but remained as micro particles (Figure 1d).

### 2.2. Density of Composites

Among the composites manufactured with low *M*_w_ PLA, the density measurement showed values of 1.0, 1,15, 1.18, 1.20, 1.42 g/cm^3^ for control, whiskers, whiskers-Zn, rods and rods-Sr filled composites, respectively. Similarly, the high *M*_w_ PLA composites showed a lower density value for control 1.10 g/cm^3^ compared to greater density values for tested composites: 1.25, 1.86, 1.40, 1.46 g/cm^3^ in whiskers, whiskers-Zn, rods and rods-Sr rich materials, respectively.

### 2.3. Distribution of Pore Size in Composites

**Figure 2:**
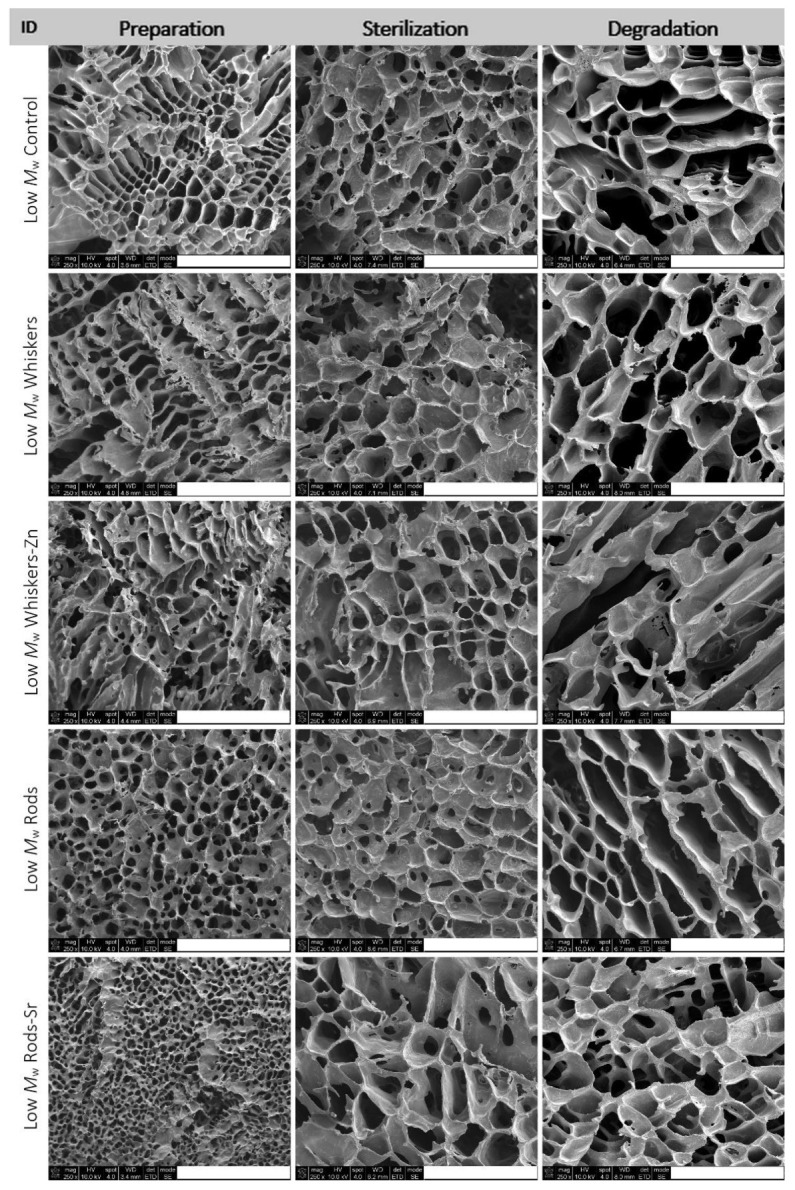
SEM images of the prepared, sterilized and degraded composites made of Low *M*_w_ PLA. Scale bar, 500 µm.

**Figure 3:**
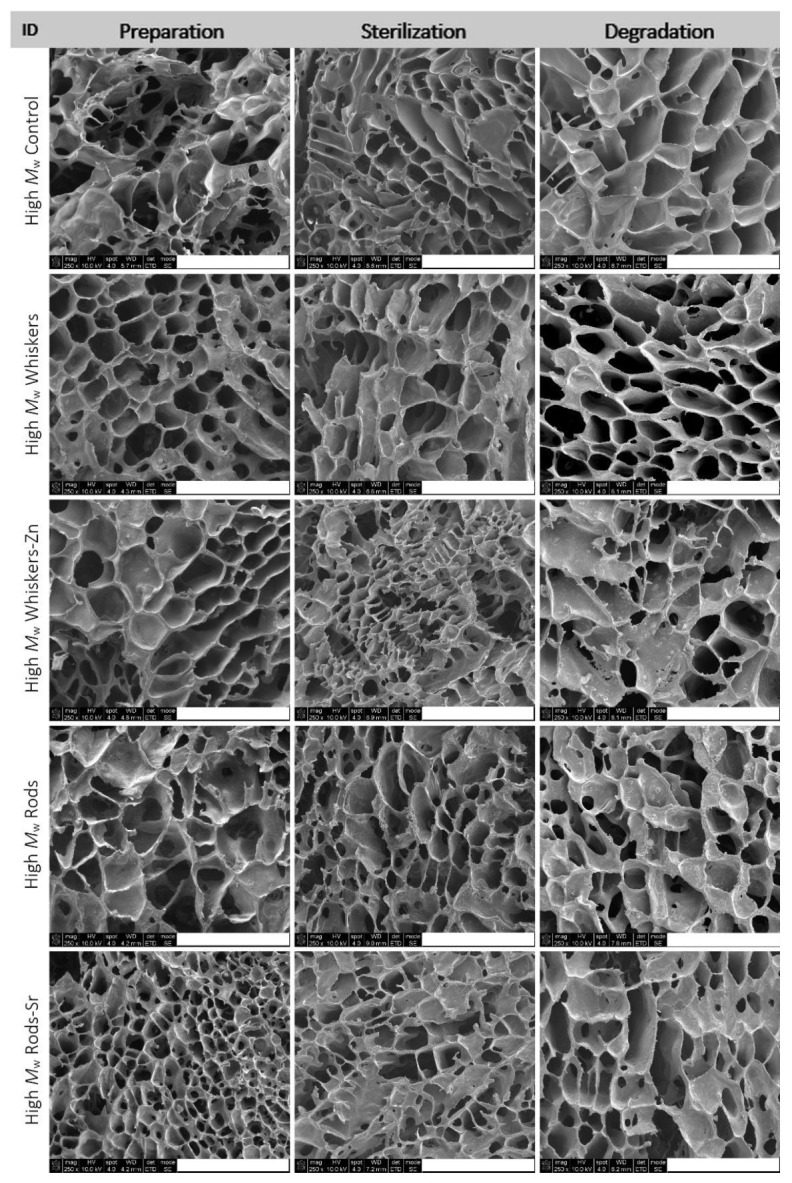
SEM images of the prepared, sterilized and degraded composites made of High *M*_w_ PLA. Scale bar, 500 µm.

The analysis of pore size distribution was conducted on the basis of SEM observation (Figure 2 and Figure 3) of prepared, sterilized composites and composites subjected to an in vitro degradation assay for 12 weeks.

**Figure 4:**
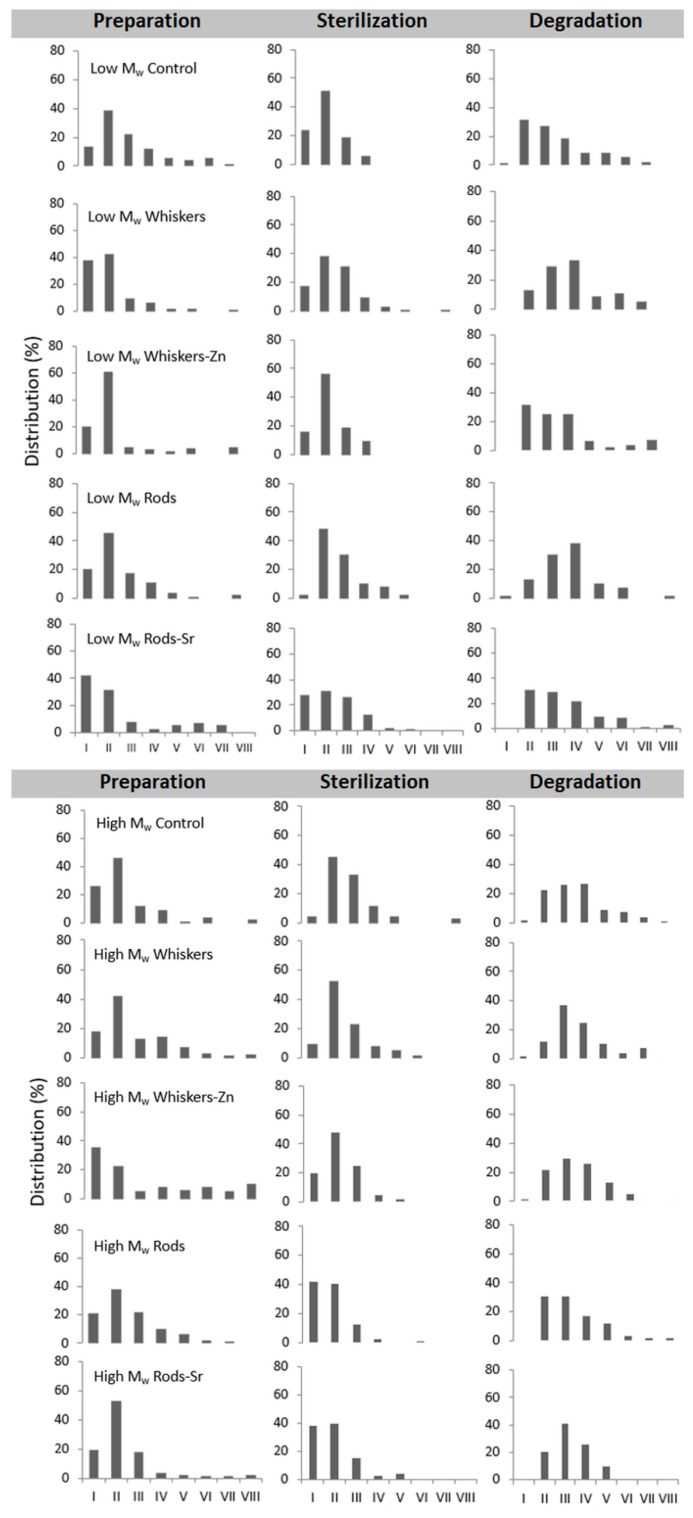
Distribution of pore size in the composites (%). *X*-axis indicates the compartment of pore size as such; I, 0–50 µm; II, 50–100 µm; III, 100–150 µm; IV, 150–200 µm; V, 200–250 µm; VI, 250–300 µm; VII, 300–350 µm; VIII, 350–600 µm.

In low *M*_w_ PLA composites, the pore size distribution of prepared composites ranged from 20 µm to 388 µm in the control (Figure 4). Addition of whiskers, whiskers-Zn and rods led to an increase in proportion of pore size in the range of 0–50 µm and 50–100 µm in composite, whereas the inclusion of rods-Sr caused an increase in the proportion of pores between 0–50 µm but slightly reduced pores between 50–100 µm, compared to the control. In high *M*_w_ PLA materials, the proportion of pore size between 0–50 µm was slightly lower in whiskers, rods and rods-Sr rich composites, but was higher in whiskers-Zn composite, compared to the control. The proportion of 50–100 µm pores of whiskers-Zn rich composites was reduced by more than half compared to the control, whereas overall this proportion of pore size was maintained about at 40% or above for control, whiskers, rods or rods-Sr composites.

The sterilization changed the pore size distribution in the low and high *M*_w_ composites. In low *M*_w_ materials, sterilization of the control sample led to an increase in the proportion of pore size between 0–50 µm and 50–100 µm. However, the proportion of 0–50 µm pores reduced in HA rich composites compared to their counterparts at preparation stage. Addition of whiskers and whiskers-Zn, furthermore, led to a slight reduction of the concentration of 50–100 µm pores, but inclusion of rods and rods-Sr remained this pores concentration at the similar range in the sterilized stage as in their counterparts of preparation products. In high *M*_w_ composites, the pore size of 0–50 µm was reduced after sterilization of control, whiskers and whisker-Zn composites but increased in rods and rods-Sr composites compared to their counterparts in the preparation stage. The addition of whiskers and whiskers-Zn, furthermore, led to an increase in fraction of pore size 50–100 µm in the sterilized materials.

Both for low and high *M*_w_ PLA composites, the period of 12 weeks incubation caused an increase in pore size in control and tested materials; it was worth to notice that the proportion of pore size 0–50 µm almost disappeared in all composites.

### 2.4. Molecular Properties

The *M*_w_ of low *M*_w_ starting polymer (826,576 Da) was reduced on average by 22% during the preparation of composites, whereas in the case of the high *M*_w_ polymer (1,471,000 Da) the same process reduced *M*_w_ by 30%. Simultaneously, the dispersity index of PLA raised by 28% and 25% in low and high *M*_w_ composites, respectively, compared to the unprocessed polymer counterparts. The sterilization led to further reduction of *M*_w_ on average by 76% and 84% in low and high *M*_w_ materials, respectively compared to the polymer *M*_w_ in prepared composites. However, the dispersity indices raised only on average by 12% and 16%. The 12 weeks of degradation caused an additional reduction of *M*_w_ by 24% and 21% in both low and high *M*_w_ materials, respectively. There was no noticeable effect of HA morphology on polymer *M*_w_ changes during preparation, sterilization or degradation steps (Table 1).

### 2.5. Compression Strength

**Figure 5:**
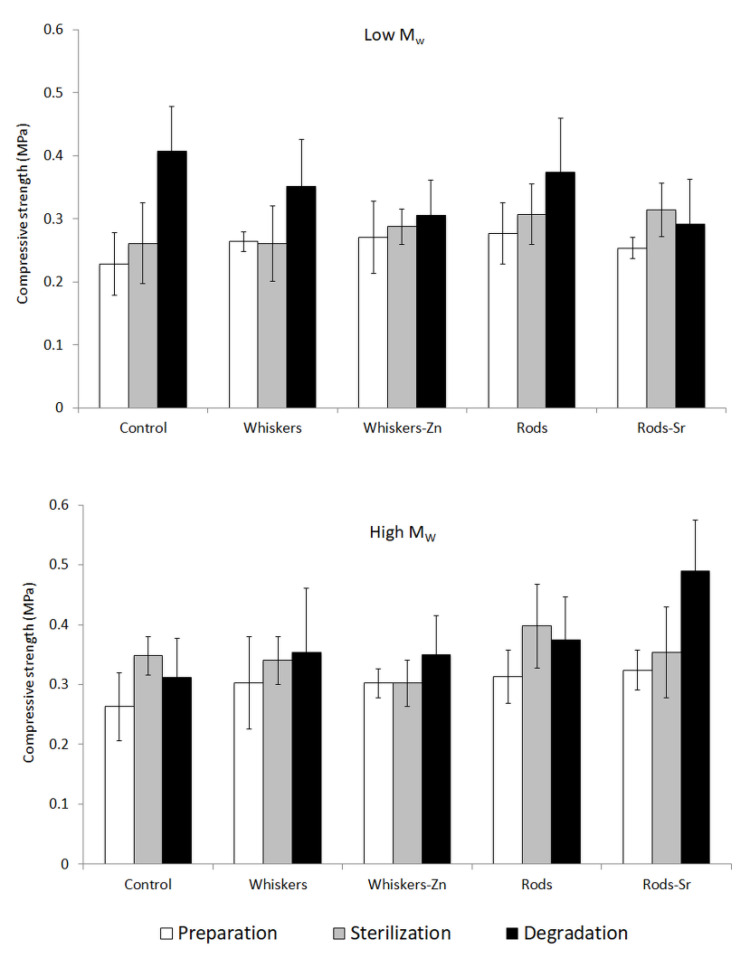
Compression strength of low and high *M*_w_ PLA composites.

The compression strength of low *M*_w_ PLA materials increased starting from the preparation step through sterilization to degradation after 12 weeks in PBS (Figure 5). An exception occurred in a degraded composite comprising Sr-rich HA, however, the standard deviation was higher than the prepared and sterilized counterparts, suggesting a similar raising trend.

Among the high *M*_w_ PLA composites the raising trend was observed only for composites with Sr rods included or whiskers added alone (Figure 5). In the case of other samples, there was impossible to determined clear dependence between compressive strength and the process step. With respect to the range of values of the standard deviation, one can say that the compression strength was very similar prior and after sterilization and degradation in the control, whiskers, whisker-Zn or rods filled composites.

### 2.6. Thermal Properties

**Figure A6:**
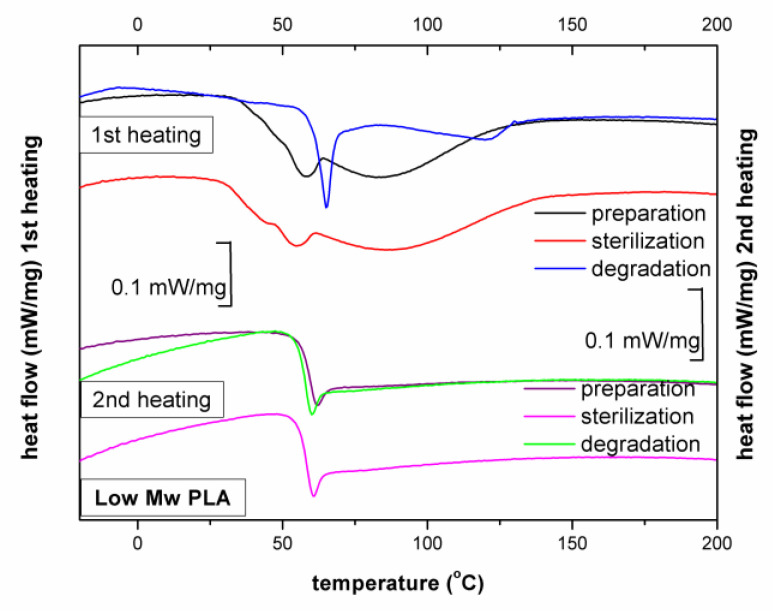
DSC curves for Low *M*_w_ PLA at different stage of studies—after preparation, after sterilization and degradation.

**Figure A7:**
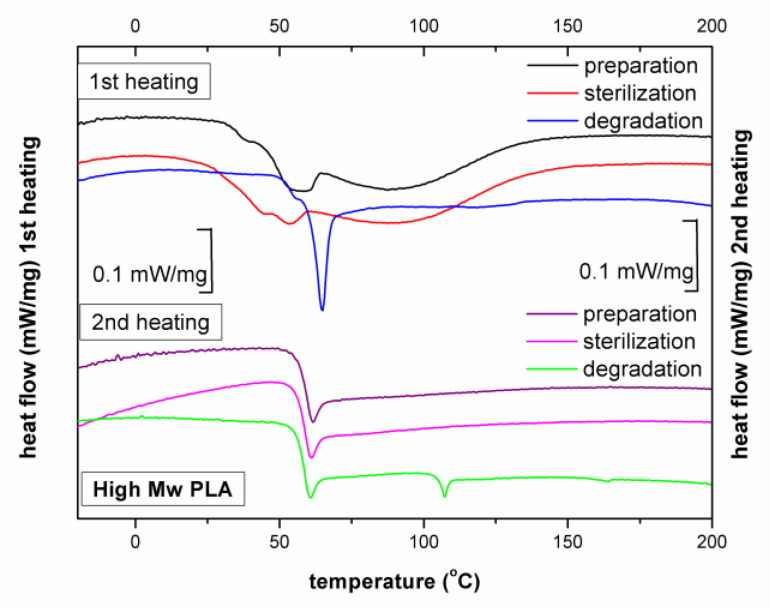
DSC curves for High *M*_w_ PLA at different stages of studies—after preparation, sterilization and degradation.

**Figure A8:**
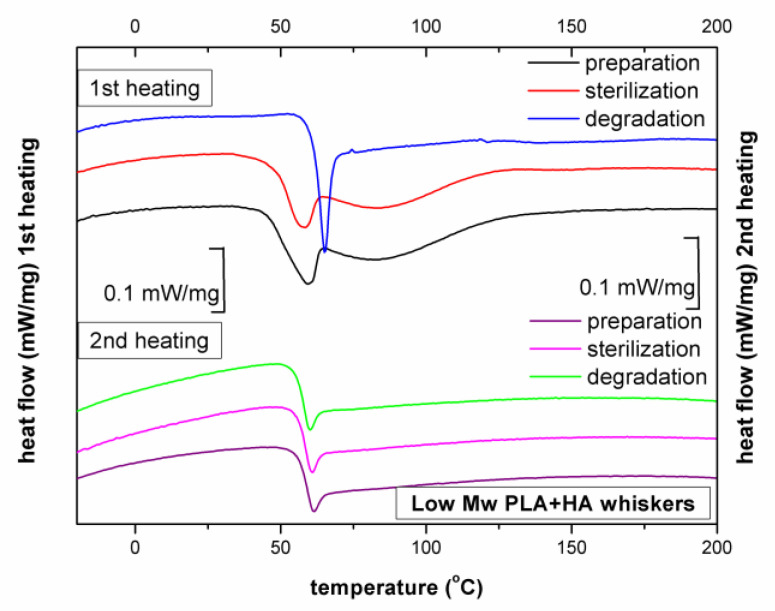
DSC curves for Low *M*_w_ PLA + HA whiskers composites at different stages of studies—after preparation, sterilization and degradation.

**Figure A9:**
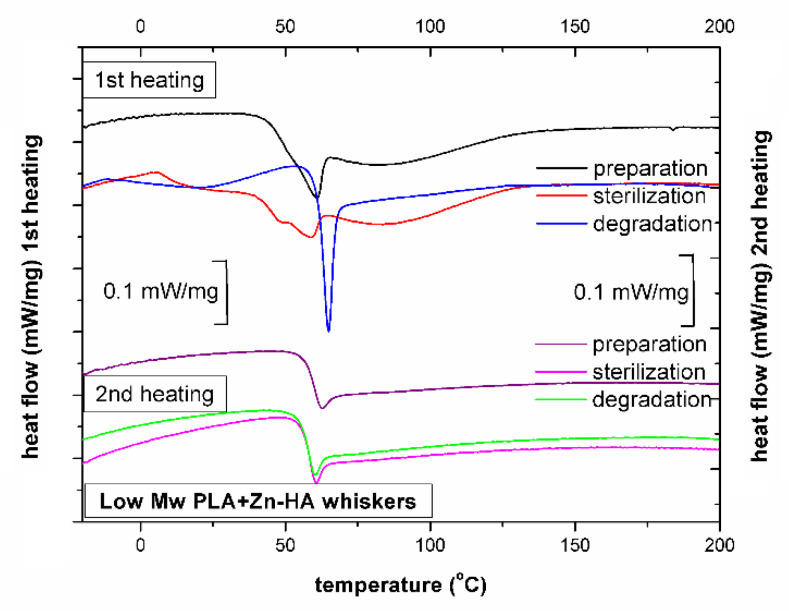
DSC curves for Low *M*_w_ PLA + Zn-HA whiskers composites at different stage of studies—after preparation, after sterilization and degradation.

**Figure A10:**
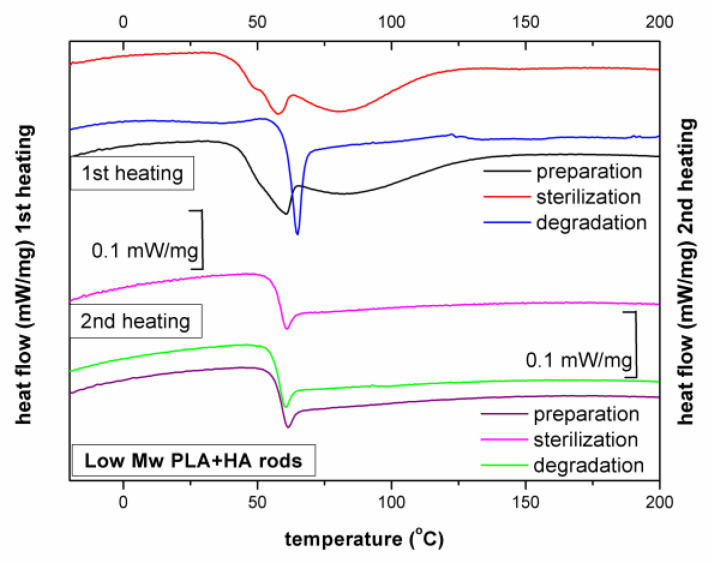
DSC curves for Low *M*_w_ PLA + HA rods composites at different stage of studies—after preparation, after sterilization and degradation.

**Figure A11:**
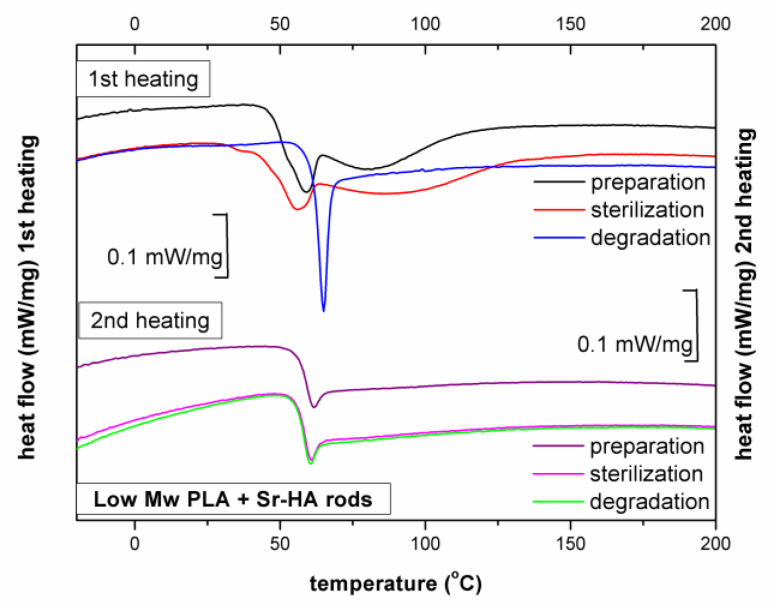
DSC curves for Low *M*_w_ PLA + Sr-HA rods composites at different stage of studies—after preparation, after sterilization and degradation.

**Figure A12:**
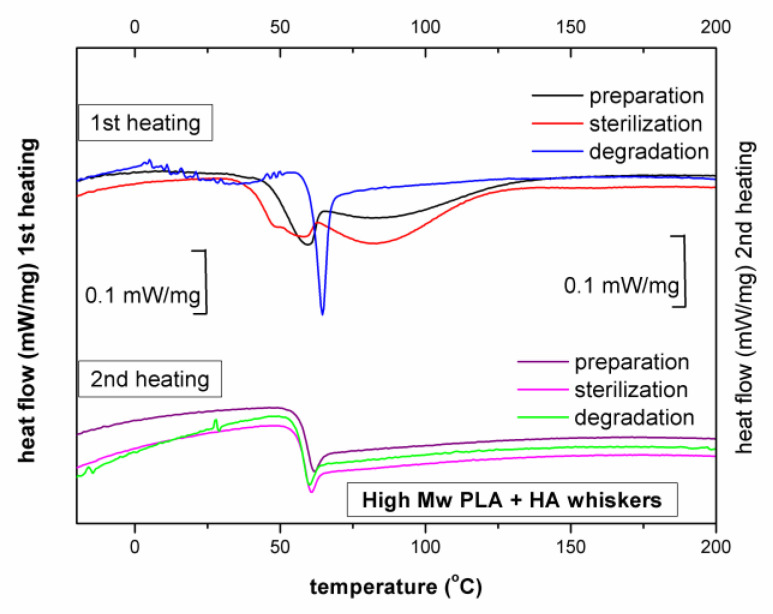
DSC curves for High *M*_w_ PLA + HA whiskers composites at different stage of studies—after preparation, after sterilization and degradation.

**Figure A13:**
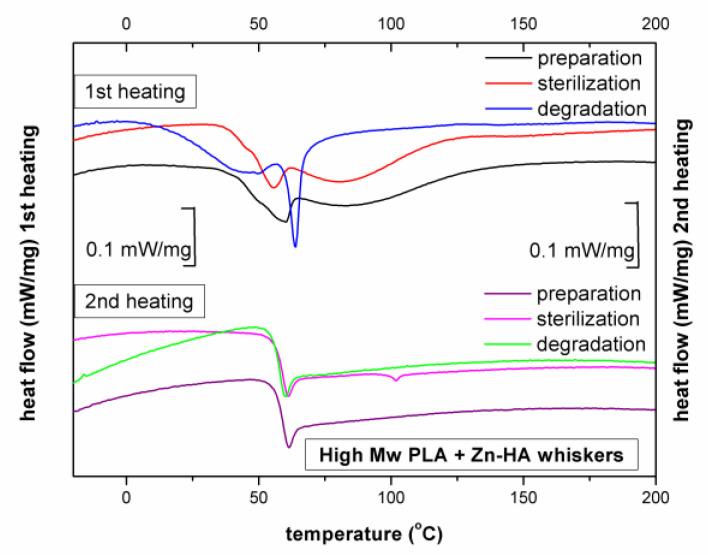
DSC curves for High *M*_w_ PLA + Zn-HA whiskers composites at different stage of studies—after preparation, after sterilization and degradation.

**Figure A14:**
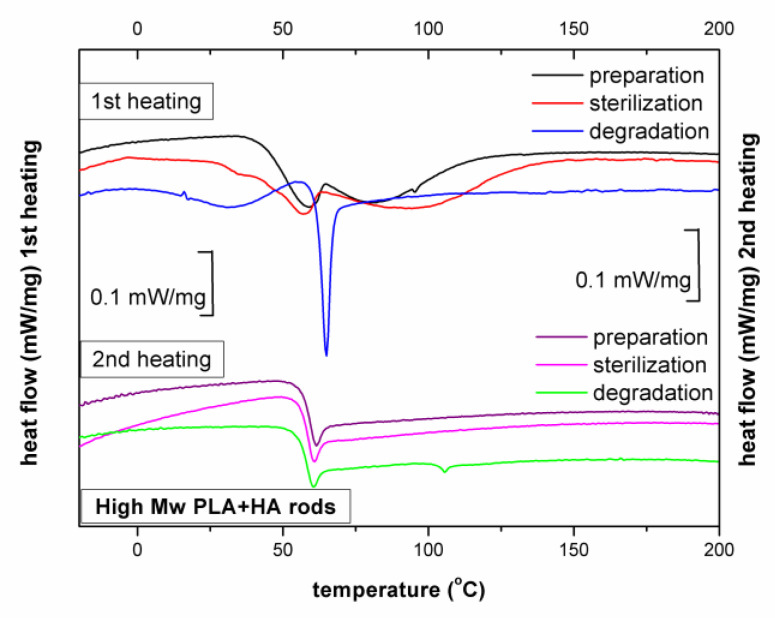
DSC curves for High *M*_w_ PLA + HA rods composites at different stage of studies—after preparation, after sterilization and degradation.

**Figure A15:**
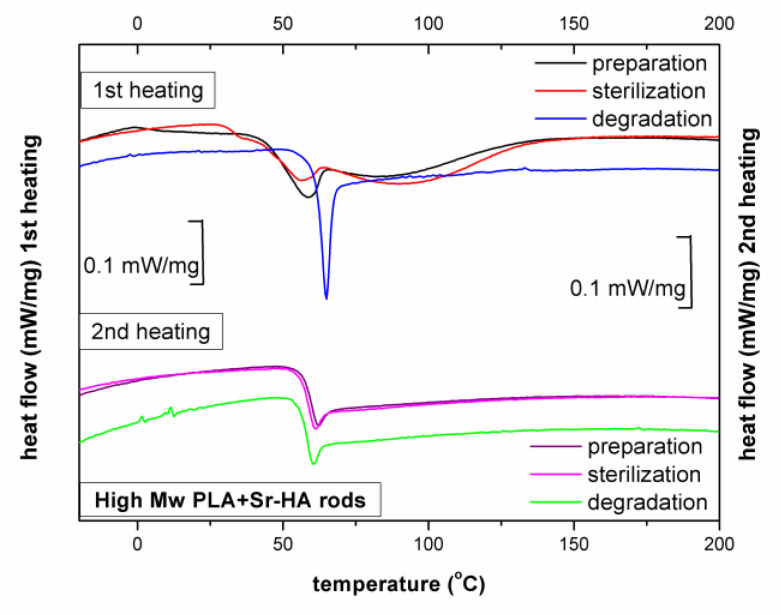
DSC curves for High *M*_w_ PLA + Sr-HA rods composites at different stage of studies—after preparation, after sterilization and degradation.

In the first heating, DSC data showed endothermal signals that might correspond to the partial melting of PLA (data showed in Appendix A—Figure A5, Figure A6, Figure A7, Figure A8, Figure A9, Figure A10, Figure A11, Figure A12, Figure A13, Figure A14 and Figure A15). It was found that for solid samples of neat PLA before any processing the enthalpy of melting was 10.70 and 12.96 J/g for low and high *M*_w_ polymers, respectively. In the case of prepared porous samples, the enthalpies were insignificantly higher for controls of neat PLA, whereas for porous composites it was in the range of 4.3–14.1 J/g with average of 9.2 J/g, resulting without obvious correlation between enthalpy, *M*_w_ of PLA or type of HA added. The process of sterilization of composites did not affect these values strongly and one can see the average increase in 1 J/g in the melting enthalpy. No clear dependence between degree of crystallinity and the stage of composites study was observed. The values of enthalpy cannot be determined correctly as no clear baseline was found, and both the enthalpy and calculated degree of the crystallinity (not presented) should be treated as the estimation. In our opinion the degree of crystallinity in the case of these materials was relatively low and similar to each other, thus it should not significantly affected the properties of the composites. It is worth to mention that after 12 weeks of degradation the all samples studied became totally amorphous, as no thermal effects was observed except of glass transition. It is postulated that after preparation of composites a part of crystallites still exist, however under controlled heating during DSC measurement no crystal formation occurs. Thus on second heating scan only *T*g can be observed.

**Figure 6:**
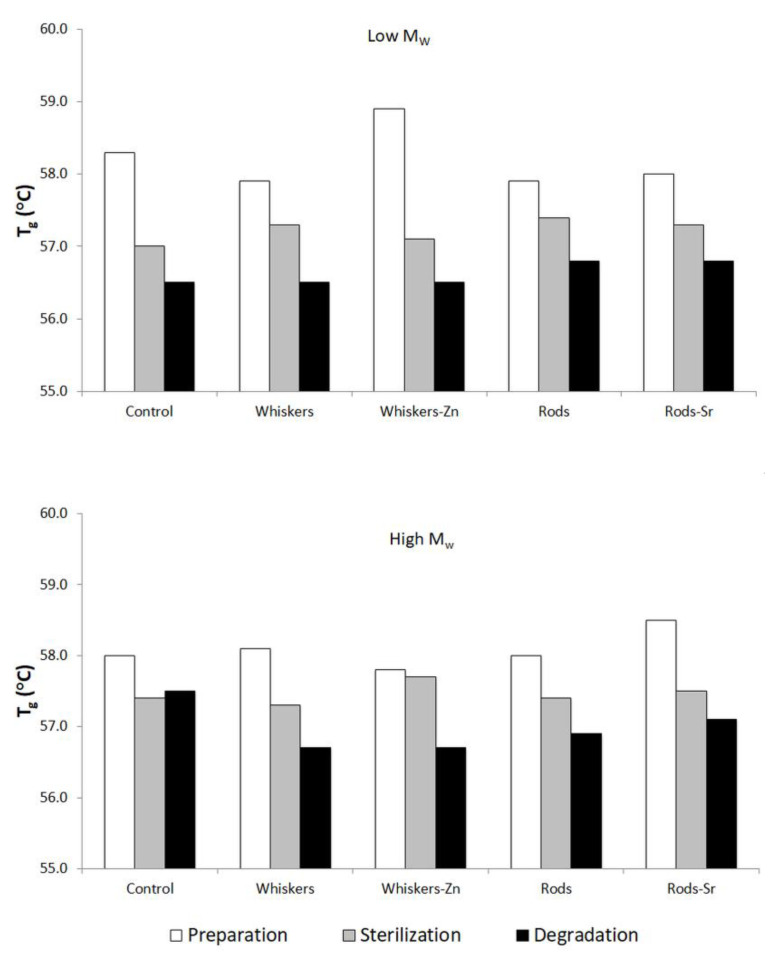
Glass transition temperature recorded for the tested composites.

DSC data of second heating in pure PLA polymers, prepared, sterilized and degraded composites showed only a peak of glass transition temperature (*T*g) in the thermograms (Figure 6). Pure low and high *M*_w_ PLA were characterized by *T*g of 58.2 °C and 58.4 °C, respectively.

Figure 6 showed the *T*g change from prepared, sterilized to degraded composite. In both low and high *M*_w_ composite, the *T*g was the greatest in the prepared materials followed by reduction in sterilized and the degraded counterparts, with an exception of high *M*_w_ control. The lowering of *T*g followed a decrease in molecular weight.

### 2.7. Weight Loss in Composites

**Figure 7:**
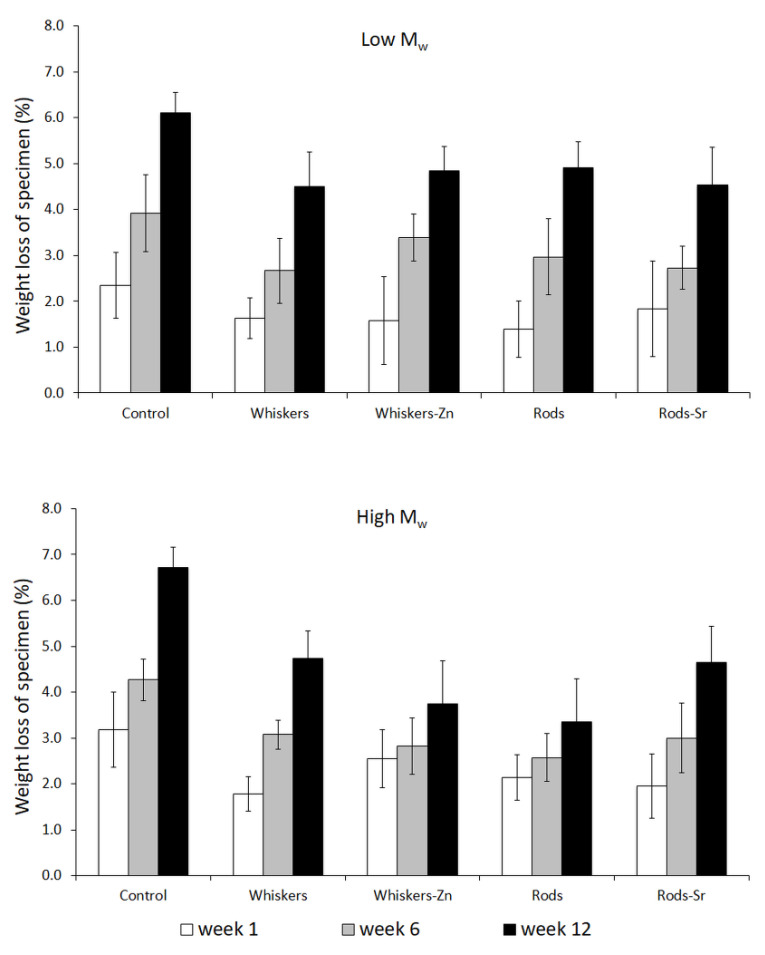
Weight loss of specimen at particular time of degradation.

The designed incubation of composites in PBS mimics the conditions inside of human body, which might help to estimate the time of scaffold degradation. Figure 7 showed the loss of materials’ weight after particular time of incubation; it is clear to notice that there was a lower reduction of sample weight in the HA rich composites in low and high *M*_w_ sets, irrespectively to the HA morphology, compared to a greater reduction in the control samples.

**Figure 8:**
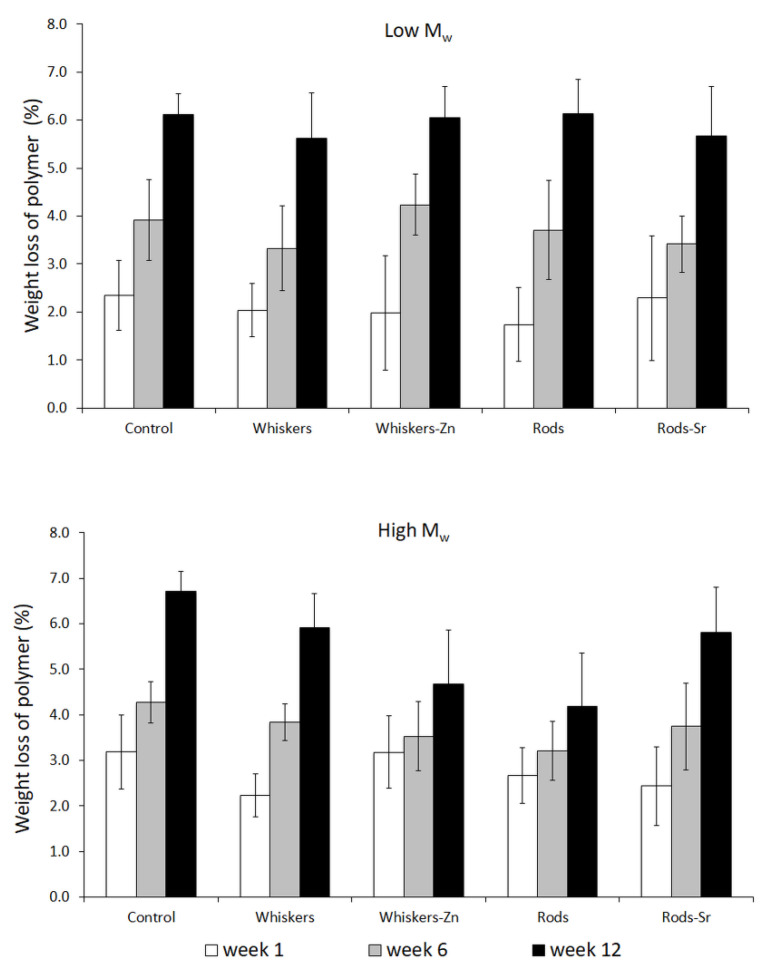
Weight loss of polymer inside composites at particular time of degradation.

Similarly, the higher reduction of polymer weight was observed for low and high *M*_w_ composites (Figure 8). Among the low *M*_w_ set, there was no clear impact of HA addition and its morphology on the polymer weight loss. However, in the high *M*_w_ materials, there was observed a slightly lower polymer loss in HA filled composites, especially in whisker-Zn and rod filled composites after 12 weeks of incubation.

### 2.8. In Vitro Cytobiocompatibility of Produced Composites

**Figure 9:**
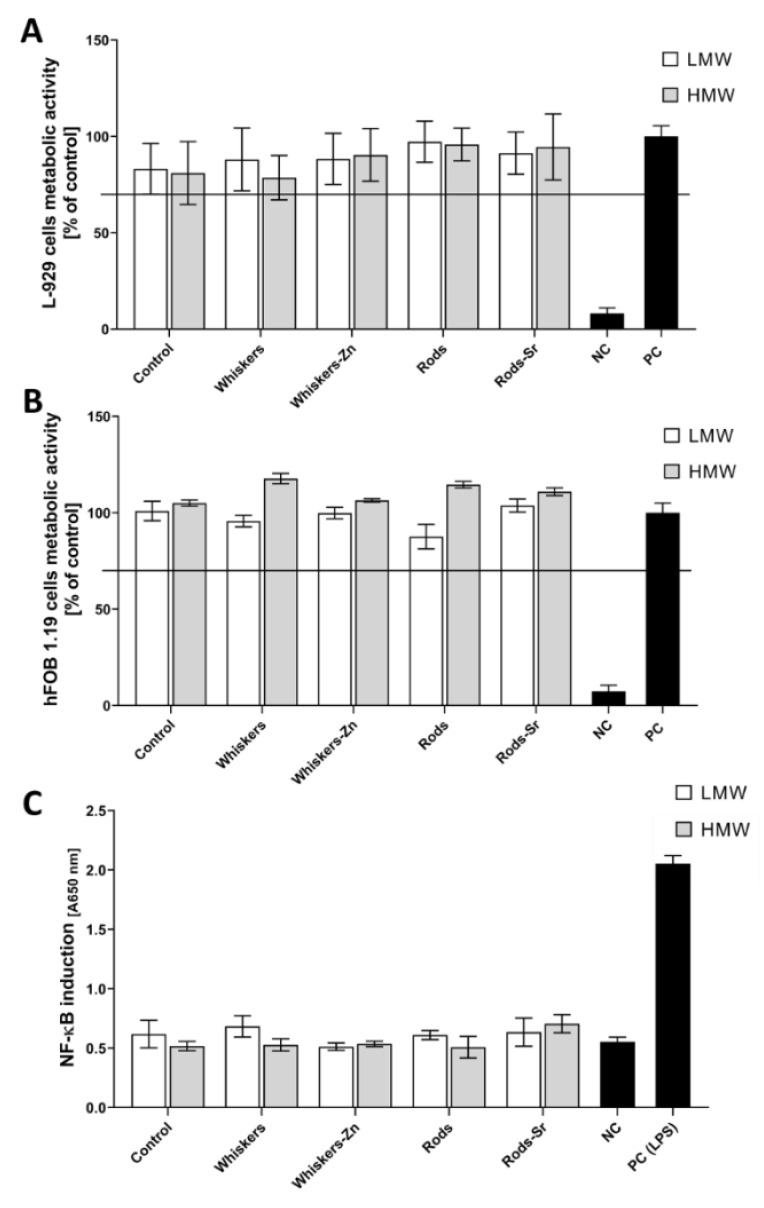
Cytobiocompatibility assessment with cells cultured on examined composites. Mouse skin L929 fibroblasts (**A**) or human hFOB1.19 osteoblasts (**B**) were co-cultured with the sections of composites according to ISO-10993-5:2009 and the viability was estimated in the MTT (3-(4,5-dimethylthiazol-2-yl)-2,5-diphenyltetrazolium bromide) reduction assay. Dotted line indicates the cytotoxicity threshold as cell viability of 70%, respective to control cells cultured without the composite (100%). The quantitative measurement of activation of the nuclear factor NF-κB in THP1-Blue™ monocytes co-cultured with composites for 24 h (**C**), in comparison to lipopolysaccharide-treated (LPS *Escherichia coli* O55:B5, positive control) or non-treated cells (culture medium, negative control). The toll-like receptor-mediated activation of NF-κB resulted in the production of secreted embryonic alkaline phosphatase (SAEP) which was assayed in a colorimetric reaction (absorbance at 650 nm) with QUANTI-Blue™ substrate solution. Data are presented as mean ± SD of three separate experiments.

The viability of cells cultured in the presence of tested composites was not significantly affected by the biomaterials. We can conclude that all produced composites met the criterion of ISO 10993-5:2009 document, which states that the biomaterials that did affect the cell viability below 70% should be recognized as non-cytotoxic, and may be further tested in more complex in vitro experiments (Figure 9A,B). In the case of hFOB1.19 cells, there was a trend towards improved cell viability or increased metabolic activity in the presence of high *M*_w_ polymer containing scaffolds comparing to low *M*_w_ polymer-containing materials. Importantly, the biomaterials can be considered non-pyrogenic as they did not induce the toll-like receptor-mediated activation of NF-κB factor in THP1-Blue™ monocytes (Figure 9C).

## 3. Discussion

### 3.1. Preparation

An ideal absorbable composite for bone fracture fixation should have an appropriate porosity after preparation and sterilization and remaining strength in the course of composite degradation, as long as the healing fracture needs support [29]. In this paper, to remain the mechanical properties of composites, we prepared two morphological different forms of HA i.e., hexagonal rods and whiskers and included into the matrix composites of low and high *M*_w_ polymer, using a thermally induced phase separation method. The porosity and the size of pores in composites should mimic the natural bone extracellular matrix. Microstructure, one of the most important feature of scaffold is to ensure the cell migration and transport of nutrients. The average size of pores is required to be in a range of 100–350 µm for the regeneration of bone tissue [30]. In our study, the prepared composites had a distribution of pore size in a range of 50 µm to 600 µm compartments that is in line with addressed needs for location and function of bone tissue.

The shape of the scaffold determines its mechanical properties, but, furthermore, the inclusion of fillers has an impact on the structural integrity. The compressive strength of cylindrical specimens was in a range of 0.2–0.3 MPa. In low and high *M*_w_ PLA prepared products, the inclusion of HA insubstantially increased the compressive strength in the composites compared to the controls. However, there was no pronounced influence of HA morphology or metal addition on the mechanical property.

### 3.2. Sterilization

A fundamental step in the manufacturing process of biomaterials is sterilization in order to avoid any complications such as infections or rejections [31]. The intensive progress on development of biofunctional materials have been continuously growing over the last years, but the sterilization methods remained mainly unchanged. Besides the three most industrially used methods such as ethylene oxide treatment, gamma irradiation and steam sterilization, there are, furthermore, dry heat sterilization, peracetic acid treatment, electron beam (E-beam) or H_2_O_2_ gas plasma sterilization. Both heat methods (i.e., steam and dry heat) are not suitable for heat sensitive materials. In chemical treatments, ethylene oxide and peracetic acid might be either toxic and cause carcinogenic outputs. Application of H_2_O_2_ gas plasma might cause not only changes in chemical and mechanical properties of polymers but, furthermore, might induce formation of the reactive residuals. The irradiation techniques such as gamma and E-beam, offer a low temperature and short processing time with a range of penetration ability.

The gamma radiation uses high-energy photons emitted by a ^60^Co source and have no mass or charge, whereas the E-beam sterilization uses fast electrons (with both mass and charge) that has a lower penetration ability compare to gamma treatment [32]. However, in practice, the gamma irradiators deliver dose rate between 2 and 10 kGy per hour, while the E-beam accelerators can deliver dose rates more effectively by a factor of 1000 [20]. Therefore, greater dosing rate of E-beam causes less expose time and reduced potential degradation of polymers. One of the main advantages is the high-energy power of E-beam can sterilize in seconds.

Although the gamma radiation conditions with a dose level of 25 kGy became recommended by the International Organization for Standardization (ISO) for health care products, some research showed that this condition can, furthermore, cause degradation of polymers. A study by Soriano et al. [33] examined the influence of gamma sterilization on PLA and found a reduction in *M*_w_ by 26–35% compared to the untreated sample. Thus, the selection of treatment parameters is of great importance. The impact of irradiation method on biomaterials is affected by the exposure time, penetration depth and dose rate. As PLA is a thermal and hydrolytic sensitive material, conventional sterilization methods may cause the physical and molecular degradation of the polymer. In the current study, we selected the E-beam radiation within the irradiation methods in order to ensure short exposure time and test the effect of this type of sterilization on the properties of PLA composites.

However, in our examined samples, the *M*_w_ of both polymers substantially reduced when the E-beam irradiation was applied, compared to their preparation counterparts. Despite of the advantages of this type of sterilization, literature, furthermore, mentioned that the E-beam might lead to degradation of polymers by cross-linking, chain scission or a combination of both, depending on type of polymer tested [21,22]. The chain scission might occur as random rupturing in weak bonds of polymer that results in a reduction of its molecular weight and formation of shorter chains, which might go further reduction in molecular size [34]. The mechanism of crosslinking can undergo towards the formation of large three-dimensional network that further might translate into cracking, discolouration, brittleness or intensive polymer degradation [35] but, furthermore, might improve the mechanical properties of composite. The examined samples in this publication showed a slightly greater compression strength after sterilization irrespectively to the HA inclusion or its morphology/metal doping, which might be a result of occurrence of crosslinking.

The porosity of PLA composites might, furthermore, be affected by the sterilization conditions. A study by Türker et al. [36] showed that the application of irradiation-based sterilization method caused a decrease in pore size in PLA. Similarly, Torres-Giner et al. [37] reported the reduction of pore size in PLA due to radiation sterilization. Both studies indicated, furthermore, an increase in surface roughness as a result of substantial scission of polymeric chains due to radical formation; this phenomenon might explain some changes in the distribution of pores in our tested composites.

Although the E-beam is considered as safe to health products, some changes in pore distribution and compressive strength were observed. More importantly, this sterilization procedure caused the substantial reduction of *M*_w_ in PLA (as clearly confirmed by GPC data), which might have further consequences in degradation of composites and mechanical properties in the extended period of incubation by in vivo design. The scission mechanism is predominant, thus PLA macromolecules present in composites undergo partial degradation. Therefore, it is recommended to select the adjusted condition to maintain the physicochemical and structural integrity of composite or take into an account changes occurred during the sterilization prior to implantation.

### 3.3. Degradation

Degradation behaviour of biodegradable materials is a critical factor affecting healing of bone fracture. Similar to sterilization, in the course of degradation, the PLA molecules undergo chemical reactions such as bond cleavage, chain crosslinking but, furthermore, oxidation when exposed to UV light, air or oxygen [38]. As a result of this degradation, on the macro level, the PLA rich composite might show deformation or brittle fraction that affect its physicochemical properties. A number of studies investigated the degradation of PLA based composites comprising HA, however, they show conflicting outcome of the impact of bioceramics on degradation mechanism.

Delabarde et al. [23] showed that inclusion of HA into PLA matrix could accelerate degradation at the interface of polymer matrix/particles. Similarly, studies by Ignatius et al. [24] and Navarro et al. [25] reported that the inclusion of beta-TCP or soluble calcium phosphate glass led to an increase the degradation rate of PLA; this increase in degradation of PLA with the presence of Ca-P fillers might be due to the hydrophilicity of the fillers and their structural arrangement in the particle/matrix interface [26]. In our study, the distribution of pores in composites substantially increased after incubation for 12 weeks, which was a result of substantial degradation of *M*_w_ and weight loss of composites.

On the other hand, Bleach et al. [39] reported that the PLA composites without inclusion of fillers enabled absorption more water and showed greater loss in its weight compared to the composites with included HA or tricalcium phosphate (TCP) fillers, when immersing the materials in simulated body fluid (SBF) for 12 weeks. Similarly, a study by Niemelä [27] showed a slower degradation of composite composed of PLA and beta-TCP compared to unfilled PLA; this might be due to the fact that mineral incorporation into PLA composite improved the thermal stability of polymer as suggested by Araújo et al. [40]. Besides reduction of *M*_w_, our study showed that inclusion of HA led to reduction of composites weight loss in the course of incubation; this indicates that the incorporation of HA in a form of whiskers or hexagonal rods into polymer matrix might prevent its degradation.

In the course of degradation, the thermal properties of PLA composites were assessed. The *T*g reduction from pure polymer to a form of degraded composite was the results of polymeric chain cleavage [41]. Given the fact that above *T*g the amorphous polymer (phase) reaches viscoelastic state in which the fragments of polymer chains are able to change its conformation, the longer polymer chain the *T*g is greater. Thus, the shorter chains formed in the course of degradation require lower temperature to gain ability for the chain movements that is translated into reduction of *T*g. In contrast to some reports [28,42], our polymers showed no cold crystallization behaviour in the course of degradation. In the second heating, DSC showed only one *T*g peak in all tested materials, indicating that the amorphous structure was formed.

In terms of the compressive strength of our tested composites, it is noticed that the property sometimes raised after degradation compared to their starting counterparts, although it was not substantially change. A study by Wang et al. [26] investigated the degradation of composites made of PLA and nHA and suggested that short molecules of PLA degradation might play a role of plasticizer and improve the toughness of the composites to some extent; this might explain the variation or increase in mechanical properties of composite after degradation.

## 4. Materials and Methods

In this research, we produced HA with two types of morphology, from the following substrates: sodium dihydrogen phosphate (CAS:7558-80-7, pure p.a. Chempur, Piekary Śląskie, Poland), calcium nitrate tetrahydrate (CAS:13477-34-4, pure p.a. Chempur, Piekary Śląskie, Poland), urea (CAS: 57-13-6, pure p.a. Chempur, Piekary Śląskie, Poland), potassium hydrogen phosphate (CAS: 7758-11-4, Chempur, Piekary Śląskie, Poland), zinc nitrate (CAS:10196-18-6, Sigma Aldrich, Hamburg, Germany), strontium nitrate (CAS: 10042-76-9, Sigma Aldrich, Hamburg, Germany), calcium lactate pentahydrate (CAS: 5743-47-5, Chempur, Piekary Śląskie, Poland), nitric acid (CAS: 7664-38-2, Chempur, Piekary Śląskie, Poland) and 1,4-dioxane (CAS: 123-91-1, Merck, Darmstadt, Germany) as solvent. In addition, we purchased two types of PLA from Evonik, DE such as Resomer LR 706s and LR 708, later referred as low and high *M*_w_ polymer, respectively. For biological assays following cell lines: L-929 (CCL-1™), mouse fibroblasts (ATCC, Manassas, VA, USA), hFOB 1.19 (CRL-11372™) human fetal osteoblastic cell line (ATCC, Manassas, VA, USA) and THP1-Blue™ NF-κB cells (InvivoGen, San Diego, CA, USA), as well as Roswell Park Memorial Institute (RPMI)-1640 medium (HyClone, Cytiva, MA, USA), phenol-free Dulbecco’s modified Eagle’s medium and Ham’s 12-F medium (Gibco, Waltham, MA, USA), fetal bovine serum (FBS) (HyClone), penicillin, streptomycin, 3-(4,5-dimethylthiazol-2-yl)-2,5-diphenyltetrazolium bromide (MTT), trypsin-EDTA solution (Gibco, Waltham, MA, USA), 4-(2-hydroxyethyl)-1-piperazineethanesulfonic acid (HEPES), blasticidin and QUANTI-Blue™ (InvivoGen) were used.

### 4.1. Synthesis and Modification of HA

The synthesis of HA whiskers was conducted using homogenous precipitation method with urea. The main substrates used for fabrication of the HA with formulate of Ca_10_(PO_4_)_6_(OH)_2_ were: sodium dihydrogen phosphate (0.12 M) and calcium nitrate tetrahydrate (0.2 M) and urea (0.16 M) for pH control. Briefly, the appropriate quantities of substrates with Ca/P molar ratio of ≅1.67 were mixed with redistilled water (conductivity ≤ 0.06 µS) in a three-necked flask with a reflux condenser attached and heat treated at 90 °C for 48 h. Finally, the obtained precipitated solid phase was washed with boiling distilled water and dried at 90 °C for 48 h. The doping of HA whiskers with zinc occurred in the course of synthesis in the reference procedure by addition of zinc nitrate hexahydrate in a forty-sixth hour of the reaction and was further continued for the next 2 h to completion.

Synthesis of HA hexagonal rods was conducted by the method of chelate decomposition. The main substrates were: potassium hydrogen phosphate (0.12 M), calcium lactate pentahydrate (0.2 M) and nitric acid which were mixed in hydrothermal reactor at 200 °C for 5 h with continuous mixing, followed by water washing and drying at 90 °C for 24 h. Modification of hexagonal rods occurred after synthesis by mixing produced HA with strontium nitrate at 50 °C for 16 h. Similarly, to the previous procedures, the obtained material was washed and dried.

### 4.2. Formulation of Composites

Ten porous composites consisting of PLA with and without addition of HA were prepared by thermally induced phase separation method (Table 2). Shortly, the appropriate amount of individual produced HA was weighted into glass tubes and filled with solvent 1,4-dioxane, followed by mixing at sonification bath for 15 min. Mixing at sonification bath was proved in our laboratory to prevent aggregation of HA in the course of preparation of composite. Afterwards, the appropriate amount of polymer in the pellets form was added and mixed for 72 h in room temperature with HA in 1,4-dioxane dispersion until dissolved and then lyophilized including washing stages in order to remove the solvent in the prepared materials. The obtained cylindrical specimens have a height of 10 mm (±2.00 mm) and diameter of 13 mm (±1.00 mm), were further characterized.

### 4.3. Sterilization

Prior to further analysis, the composites were subjected to a sterilization with fast electron radiation at Institute of Chemistry and Nuclear Technology, Warsaw, Poland. The set dose of radiation was 25 kGy, the speed of the transporter 0.462 m/min, and the set current 600 mA; this process was intended to destroy microbial contamination. The obtained composites were further analyzed for microstructure, molecular weight and compressive strength.

### 4.4. Hydrolytic Degradation

The prepared composites were weighed on analytical balance and for each type of composite material 7 specimens were immersed in phosphate-buffered saline (PBS) in 15 mL plastic tube with an addition of 0.1 M sodium azide to prevent microbial growth. The samples were incubated at 37 °C for 12 weeks in a heater. The PBS solution was replaced once a week in the course of degradation process. Samples were withdrawn at week 1, 6 or 12, followed by washing with deionized water and lyophilized in a Beta 1-16 Christ freeze-dryer, Martin Christ Gefriertrocknungsanlagen GmbG (Osterode, Germany). Afterwards, composites were again weighed and subjected to further analysis. The degree of degradation was determined by measuring the weight loss, change in microstructure, molecular weight and compressive strength.

### 4.5. Density

The absolute density of composites was determined by the pycnometric method using a helium pycnometer (Ultrapyc 1200e, Quantachrome, Boynton Beach, FL, USA). Briefly, samples were weighed and placed in the chamber. Subsequently, chamber was filled with helium. Analysis in the helium atmosphere enabled optimal penetration of the finest pores and thus precise determination of the volume and then calculation of density. Analysis was conducted with five replicates, with a mean and standard deviation reported.

### 4.6. SEM

Analysis of produced HA and composites was conducted with scanning electron microscopy with emission scanning Nova NanoSEM 200, FEI (Eindhoven, The Netherlands). Prior to measurements, the samples were coated with gold layer using sprayer Leica EM SCD500 (Vienna, Austria) in order to ensure conductivity of the surface as well as protect the samples against electron beam. Analysis of microstructure was investigated under high vacuum conditions using an ETD detector at accelerating voltage of 10 kV.

Image analysis software (ImageJ, NIH, Bethesda, Rockville, MD, USA) was further used to calculate the diameters of pores size in the composite and length and width of HA whiskers and hexagonal rods. Then, the distribution of pore size was completed for each of the produced composite.

### 4.7. X-ray Diffraction (XRD)

The XRD was used in order to identify the crystalline phases present in the samples after synthesis. The measurements were performed on a Bruker-AXS D8 DAVINCI diffractometer (Billerica, MA, USA) with Bragg-Brentano geometry, equipped with a LynxEye detector using Cu Kα radiation and Ni filter. Diffractograms were recorded in an angular range from 5 to 120° 2θ, with a step size of 0.01°, and an exposure time of 2 s. The crystalline phases were determined from a comparison of the registered patterns with the Crystallography Open Database (COD) using the DIFFRACplus EVA-SEARCH software.

### 4.8. Weight Loss

The weight loss of polymer in specimen was calculated with the following formula:

(1) $$U_{m}=\frac{m_{0}-m_{1}}{x\times m_{0}}\times 100\%$$

where: *U_m_*—weight loss [%], *m*_0_—weight before incubation [g], *m*_1_—weight after incubation [g], *x*—content of polymer in the specimen [%]. The weight loss of whole specimen was simply measured by subtraction of weight before and after degradation of sample, and multiplied by 100% and divided by weight before degradation.

### 4.9. Molecular Weight (Gel-Permeation Chromatography)

Samples of 3–5 mg were dissolved in dichloromethane (including 1% volume chloroform) and mixed in a shaker overnight in order to completely dissolve the polymer. Solutions were filtered through a PTFE membrane with 0.2 μm pores followed by injection to a GPC system. The system was built with Viscotek GPCmax unit (Malvern, UK) and a 305 TDA detection unit with a UV measuring cell, RI detector, hybrid Right-Angle Light Scattering/Low-Angle Light Scattering (RALS/LALS) detectors, and a viscometer. The system used a Jordi Labs DVB MB column (Mansfield, MA, USA) with mixed bed (5 µm) with dimension of 30 cm length and 7.8 mm of internal diameter. The continuous phase was dichloromethane at 30 °C with a flow rate of 1 cm^3^/min. Before the sample injections, the sharp polystyrene standards were used for calibration. The *M*_w_ and the dispersity index (*M*_w_/M_n_) of the PLA were calculated with OmniSEC software.

### 4.10. Mechanical Properties of Cylindrical Specimens

Mechanical properties of the composites were evaluated by compressive test on the Zwick Roell 5kN ProLine machine (Ulm, Germany) with TestXpert III software. Measurement was conducted with the approach speed of 2 mm/min and preload 5 N, followed by continuous measurement with speed of 0.6 mm/min to 50% deformation. The composite strength values were read at deformation of 10% and reported as a mean of five replicates.

### 4.11. Differential Scanning Calorimetry

The thermal properties of PLA/HA composites were determined by DSC204 F1 Phoenix microcalorimeter (Netzsch, Selb, Germany). The samples of composites were closed hermetically in 40 µL aluminium crucibles with hole in the lid. The analysis was conducted in the range of −30 to 200 °C with a heating rate 10 °C/min, atmosphere N_2_ at 30 mL/min with 6 steps (I: −30 °C, 5 min; II: −30 °C → 200 °C; III: 200 °C/5 min; IV: 200 °C → −30 °C; V: −30 °C/5 min; VI: −30 °C → 200 °C). Data of second heat run were reported.

### 4.12. Biological Analyses

The manufactured composites were subjected to cytotoxicity test using standard direct contact cytotoxicity assay with L929 mouse skin fibroblasts (CCL-1™), according to ISO10993-5:2009 (Biological evaluation of medical devices—Part 5: Tests for in vitro cytotoxicity). Analogous tests were, furthermore, performed using human osteoblastic cell line hFOB1.19, a model more relevant to bone regeneration as described in details elsewhere [43]. Both cell lines were obtained from the American Type Culture Collection (ATCC, Manassas, VA, USA).

In addition to cytotoxicity assessments, THP1-Blue™ NF-ĸB cells were exploited as a pyrogen responsive human monocytic cell line, designed for monitoring the NF-ĸB signal transduction pathway. The cells can detect NF-ĸB activation via Toll-like receptors and provide a unique tool for quantitative assessment of the NF-ĸB-driven, immunologically relevant responses of human monocytes in vitro. The experimental method used to detect activation of the nuclear factor NF-ĸB is described in detail elsewhere [44]. Briefly, the monocytic cells were cultured as cell suspension in RPMI 1640 medium with supplementation of heat-inactivated FBS, 2 mM glutamine, 25 mM 4-(2-hydroxyethyl)-1-piperazineethanesulfonic acid (HEPES), penicillin/streptomycin solution (100 U/mL; 100 µg/mL), and 100 µg/mL normocin at 37 °C in a humidified atmosphere enriched in 5% CO_2_. Before the experiment, cells were passaged onto the same medium with addition of 10 µg/mL blasticidin. For NF-ĸB activation, 2 × 105 cells were seeded onto each well of 96-well plate (Nunc) and sections of the biomaterials or suspensions of their constituents were added to individual wells in four replicates. 2.5 ng/mL of lipopolysaccharide from Escherichia coli serotype O55:B5 or plain medium were used as a positive or negative control, respectively. Following the exposure, the culture supernatants were mixed with the QUANTI-Blue™ substrate reagent for quantitative detection of the activity of secreted embryonic alkaline phosphatase (SEAP), induced by NF-ĸB nuclear factor. The resulting colorimetric reaction was developed for 4 h at 37 °C and measured at 650 nm using the Multiskan EX plate reader (Thermo Scientific, Waltham, MA, USA).

## 5. Conclusions

This study demonstrates the influence of sterilization and degradation process on the compressive strength, pore size distribution, weight loss and molecular properties of PLA in composites made of polymer and HA included as a form of whiskers or hexagonal rods. The PLA was in the amorphous state before and after the preparation, sterilization and degradation of composites. The main conclusions could be stated as follows:

(1) Sterilization led to a reduction of *M*_w_ of PLA and, furthermore, a change in the pore size distribution of composites depending on *M*_w_ and HA added.
(2) Similarly, degradation reduced the *M*_w_ of PLA, but did not substantially change the compressive strength of tested materials.
(3) Degradation of PLA occurred as reduction of polymer mass was greater in the control sample of high *M*_w_, compared to HA-filled materials, indicating the lower rate of PLA degradation when HA is incorporated into the PLA matrix.
(4) There was no major impact of HA morphology or metal inclusion on mechanical properties and weight loss of produced composites.
(5) All produced materials should be recognized as cytocompatible when validated against L929 mouse skin fibroblasts and hFOB 1.19 human osteoblasts. The lack of cytotoxicity was accompanied by the immunocompatibility with human monocytic cells.

## Figures and Tables

**Table 1:** *M*_w_ and dispersity index of PLA in composite after preparation, sterilization and degradation.

Sample ID	Preparation		Sterilization		Degradation	
	*M*_w_ [Dalton]	*M*_w_/M_n_	*M*_w_ [Dalton]	*M*_w_/M_n_	*M*_w_ [Dalton]	*M*_w_/M_n_
Low *M*_w_ Control	668,964	2.2	146,814	2.6	110,554	2.7
Low *M*_w_ Whiskers	657,872	2.4	141,291	2.6	116,469	3.0
Low *M*_w_ Whiskers-Zn	624,991	2.2	167,082	2.4	116,423	2.8
Low *M*_w_ Rods	638,310	2.4	156,717	2.6	120,735	3.3
Low *M*_w_ Rods-Sr	633,119	2.1	156,802	2.5	119,868	3.0
High *M*_w_ Control	1,034,000	2.3	164,076	2.5	123,425	3.5
High *M*_w_ Whiskers	1,031,000	2.5	162,828	2.7	119,578	3.3
High *M*_w_ Whiskers-Zn	1,059,000	2.5	170,110	2.5	122,374	3.0
High *M*_w_ Rods	1,142,000	2.3	167,232	2.7	121,381	2.7
High *M*_w_ Rods-Sr	1,061,000	2.9	166,265	2.7	118,867	3.4

**Table 2:** Design of experiment.

Sample ID	Concentration of Components in Composites [%Weight]					
	PLA Type		HA Type			
	LR706	LR708	Whiskers	Whiskers Zn	Hexagonal Rods	Hexagonal Rods Sr
Low *M*_w_ Control	100	-	-	-	-	-
Low *M*_w_ Whiskers	80	-	20	-	-	-
Low *M*_w_ Whiskers-Zn	80	-	-	20	-	-
Low *M*_w_ Rods	80	-	-	-	20	-
Low *M*_w_ Rods-Sr	80	-	-	-	-	20
High *M*_w_ Control	-	100	-	-	-	-
High *M*_w_ Whiskers	-	80	20	-	-	-
High *M*_w_ Whiskers-Zn	-	80	-	20	-	-
High *M*_w_ Rods	-	80	-	-	20	-
High *M*_w_ Rods-Sr	-	80	-	-	-	20

**Figure A5:**
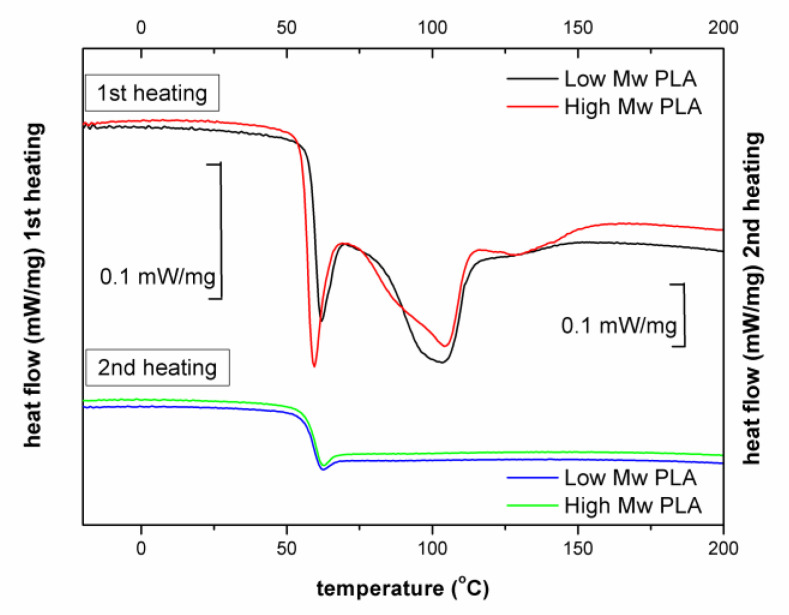
DSC curves for PLA under investigation (pure granulates).

## Data Availability

The data generated during this study are available at ŁUKASIEWICZ Research Network Institute of Ceramics and Building Materials, Center of Ceramic and Concrete in Warsaw, Biomaterials Research Group, Postępu 9, Warsaw, 02-676, Poland, and are available from the corresponding author upon request.
